# Impact of Prior mRNA COVID-19 Vaccination on PFS2 in NSCLC Patients Receiving Second-Line Immune Checkpoint Inhibitors: A Real-World Analysis

**DOI:** 10.3390/jcm15072475

**Published:** 2026-03-24

**Authors:** Selahattin Çelik, Engin Eren Kavak, Esra Zeynelgil, Gökşen İnanç İmamoğlu, İsmail Dilli, Salih Karatlı, Mehmetcan Atak, Mustafa Altınbaş, Tülay Eren

**Affiliations:** Department of Medical Oncology, Ankara Etlik City Training and Research Hospital, 06010 Ankara, Turkey; engineren2000@yahoo.com (E.E.K.); esra23.05@hotmail.com (E.Z.); gokseninanc@hotmail.com (G.İ.İ.); drdilli78@hotmail.com (İ.D.); karatlisalih@hotmail.com (S.K.); mehmetcanatakk@gmail.com (M.A.); dr.mustafaaltinbas@gmail.com (M.A.); tulayeren78@gmail.com (T.E.)

**Keywords:** non-small cell lung cancer, nivolumab, mRNA COVID-19 vaccine, immune checkpoint inhibitor, progression-free survival, immune modulation

## Abstract

**Background:** Immune checkpoint inhibitors (ICIs) targeting the PD-1 axis represent standard second-line therapy for metastatic non-small cell lung cancer (NSCLC). Emerging data suggest that SARS-CoV-2 mRNA vaccines may enhance antitumor immunity through innate immune activation and type I interferon signaling, potentially sensitizing tumors to PD-1 blockade. The clinical impact of patients initiating second-line nivolumab remains unclear. **Methods:** In this retrospective single-center cohort study, 88 patients with recurrent stage IV NSCLC who received second-line nivolumab between 1 January 2023 and 1 January 2026 were analyzed. Vaccination exposure was defined using a 6-month pre-treatment window prior to nivolumab initiation (T0). Patients were stratified according to receipt of ≥2 versus 0–1 mRNA COVID-19 vaccine doses within the 6 months preceding T0 (n = 45 and n = 43, respectively). The primary endpoint was progression-free survival from nivolumab initiation (PFS2). Survival outcomes were estimated using the Kaplan–Meier method and evaluated using Cox regression models. **Results:** With a median follow-up of 22.4 months, median PFS2 for the overall cohort was 11.1 months (95% CI, 9.4–15.1). Patients receiving ≥2 mRNA doses had significantly longer PFS2 than those receiving 0–1 dose (14.0 vs. 9.6 months; *p* = 0.04). In multivariable analysis, ≥2 doses were independently associated with reduced risk of progression or death (aHR 0.52, 95% CI 0.31–0.88; *p* = 0.01). Non-adenocarcinoma histology and baseline brain metastasis were independently associated with shorter PFS2. **Conclusions**: Receipt of ≥2 mRNA vaccine doses within 6 months before nivolumab initiation was independently associated with prolonged PFS2 in metastatic NSCLC. Prospective multicenter validation is warranted.

## 1. Introduction

Immune checkpoint inhibitors (ICIs) targeting the programmed cell death-1 (PD-1) axis have fundamentally transformed the treatment landscape of advanced non-small cell lung cancer (NSCLC) by restoring antitumor T-cell immunity and overcoming tumor-mediated immune suppression [1,2,3,4]. In patients with disease progression following platinum-based chemotherapy, nivolumab monotherapy has demonstrated a significant and durable overall survival benefit compared with docetaxel, establishing nivolumab as a standard second-line treatment option in metastatic NSCLC [1,2,5]. Despite these advances, only a subset of patients derives sustained clinical benefit from PD-1 blockade, underscoring the critical role of host immune competence, immune escape mechanisms, and tumor immune microenvironmental factors in determining therapeutic efficacy [2,6,7]. The antitumor activity of ICIs is contingent upon the presence of effective pre-existing or inducible antitumor immunity, which depends on intact antigen presentation, dendritic cell recruitment, interferon signaling, and cytotoxic T-cell priming [8,9,10]. Tumors characterized by limited immune cell infiltration, impaired antigen presentation, defective Batf3-dependent dendritic cell trafficking, and dysfunctional innate immune sensing—commonly referred to as immunologically “cold” tumors—exhibit reduced sensitivity to PD-1 blockade and inferior clinical outcomes [10,11,12]. Accumulating evidence indicates that successful immunotherapy requires coordinated activation of innate immune pathways, particularly type I interferon (IFN-I) signaling, which promotes dendritic cell maturation, antigen presentation, CD8^+^ T-cell priming, and intratumoral T-cell infiltration [13,14]. In the absence of adequate innate immune activation, adaptive immune responses remain insufficient, thereby limiting the depth, durability, and consistency of clinical benefit from immune checkpoint inhibition [2,6,15].

Messenger RNA (mRNA)-based immune stimulation has emerged as a powerful mechanism for triggering innate immune activation through pattern recognition receptor signaling, including RIG-I-like receptors and downstream interferon regulatory factor pathways, leading to IFN-I-dependent inflammatory cascades [16,17,18]. Preclinical studies have demonstrated that systemic vaccination or immunogenic RNA delivery can remodel both systemic and intratumoral immune landscapes, enhance dendritic cell activation, increase CD8^+^ T-cell infiltration, and sensitize otherwise resistant tumors to immune checkpoint blockade [19,20]. Importantly, these immunostimulatory effects are not strictly dependent on tumor-specific antigen encoding but rather on the capacity of mRNA-mediated stimulation to induce a transient, virus-like innate immune response characterized by IFN-I release and inflammatory cytokine signaling [17,19,21].

In this context, SARS-CoV-2 mRNA vaccines represent a clinically available and widely administered source of systemic mRNA-mediated immune activation [22,23]. Experimental and translational evidence has demonstrated that SARS-CoV-2 mRNA vaccination induces a robust IFN-I surge, activates antigen-presenting cells, enhances CD8^+^ T-cell priming, and increases intratumoral immune infiltration [22,24]. This immune activation is accompanied by compensatory upregulation of PD-L1 expression within the tumor microenvironment via interferon receptor-dependent signaling pathways, thereby creating a biologic state in which tumors become particularly susceptible to PD-1/PD-L1 blockade [2,24]. Notably, these immunomodulatory effects occur independently of tumor antigen specificity, highlighting a generalizable and non-tumor-specific mechanism of immune sensitization [20,22].

Emerging clinical data further support the relevance of this mechanism in cancer patients receiving ICIs. Retrospective analyses and real-world observations have demonstrated that administration of SARS-CoV-2 mRNA vaccines in temporal proximity to immune checkpoint inhibitor initiation is associated with improved survival outcomes, enhanced antitumor responses, and preserved safety profiles across multiple tumor types, including NSCLC [22,25,26]. These findings suggest that vaccine-induced innate immune activation may function as an immune primer, reinforcing antitumor immunity, enhancing interferon-driven PD-L1 expression, and ultimately augmenting the therapeutic efficacy of PD-1 blockade, particularly in immunologically cold tumors [22,25].

However, despite growing biologic plausibility and supportive clinical observations, the impact of SARS-CoV-2 mRNA vaccination on survival outcomes in patients with metastatic NSCLC treated specifically with second-line nivolumab remains insufficiently characterized [1,2,6]. Although several retrospective studies have explored the relationship between COVID-19 vaccination and immunotherapy outcomes in heterogeneous cancer populations, most included mixed tumour types or multiple immune checkpoint inhibitor (ICI) regimens and did not specifically evaluate patients receiving second-line nivolumab in metastatic NSCLC. Given the biologic dependence of nivolumab efficacy on immune activation, type I interferon signaling, antigen presentation, and PD-1 pathway engagement [7,8,10], evaluating the association between SARS-CoV-2 vaccination status and clinical outcomes in this well-defined treatment setting is of particular relevance.

To our knowledge, no prior study has specifically evaluated the association between SARS-CoV-2 mRNA vaccination and survival outcomes in patients receiving second-line nivolumab for metastatic NSCLC. Therefore, in this single-center retrospective real-world study, we aimed to investigate the relationship between SARS-CoV-2 mRNA vaccination and progression-free survival 2 (PFS2) and overall survival (OS) in patients with metastatic NSCLC receiving second-line nivolumab therapy.

## 2. Materials and Methods

### 2.1. Patient Characteristics and Data Collection

This study was designed as a retrospective, single-center cohort study to evaluate the association between prior mRNA COVID-19 vaccination and progression-free survival 2 (PFS2) in patients with recurrent non-small cell lung cancer (NSCLC) receiving second-line immune checkpoint inhibitor (ICI) therapy.

Patients diagnosed with metastatic NSCLC and treated with second-line nivolumab at the Etlik City Hospital Department of Medical Oncology between 1 January 2023 and 1 January 2026 were retrospectively screened for eligibility. Eligible patients were those who received second-line ICI monotherapy with nivolumab following documented disease progression after first-line platinum-based chemotherapy.

Inclusion and Exclusion Criteria

Inclusion Criteria

- Histologically confirmed NSCLC
- Recurrent Stage IV NSCLC
- Receipt of second-line ICI-based treatment
- Availability of reliable data on:
  ○ COVID-19 vaccination status (type, number of doses, and dates)
  ○ Treatment initiation, progression, and subsequent therapy
- Documented follow-up allowing assessment of PFS2

Exclusion Criteria

- History of another active malignancy before or during ICI therapy
- Missing or unverifiable COVID-19 vaccination records

Definition of COVID-19 mRNA Vaccination Exposure

Only mRNA-based COVID-19 vaccines (e.g., BNT162b2 or mRNA-1273) were considered. Patients who received only inactivated vaccines were categorized as non-mRNA vaccinated.

The index date (T0) was defined as the initiation date of second-line ICI therapy.

Patients were classified according to the number of mRNA vaccine doses administered within the 6 months prior to T0. The primary exposure comparison was prespecified as a binary grouping (0–1 dose vs. ≥2 doses), based on the hypothesis that receipt of at least two recent mRNA vaccine doses may generate a clinically relevant immune-priming effect before PD-1 blockade, as follows:

(1) Group A (0–1 mRNA dose):

○ No prior mRNA COVID-19 vaccination;
○ One dose of mRNA vaccine administered within the 6 months prior to T0.

(2) Group B (≥2 mRNA doses):

○ At least two doses of the mRNA COVID-19 vaccine, with the last dose administered within 6 months prior to T0.

This predefined exposure window was selected to capture the expected duration of vaccine-induced innate immune activation and immune memory expansion, which may contribute to immune priming prior to PD-1 blockade. Vaccination status was defined prior to T0 to avoid exposure misclassification after treatment initiation.

**Figure 1:**
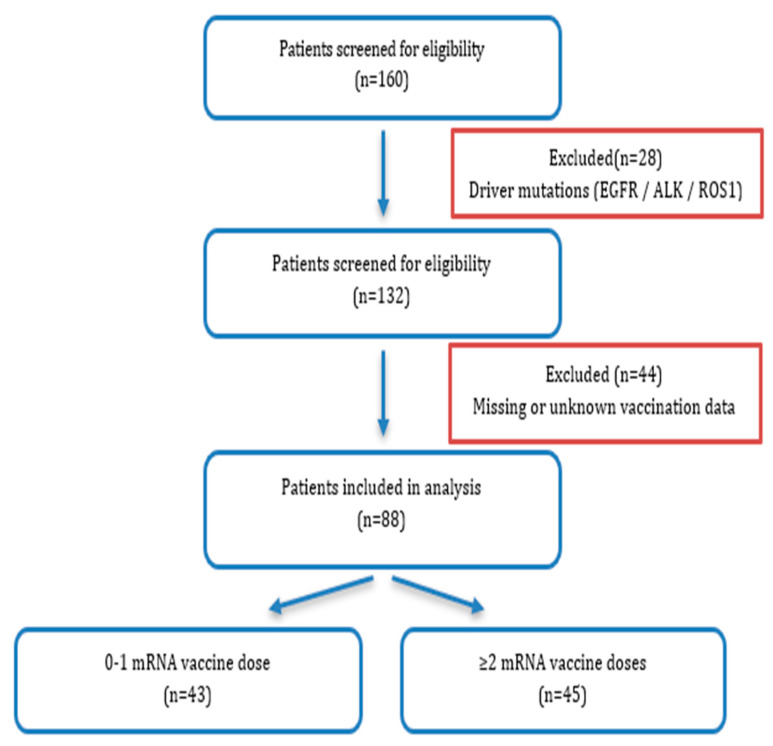
Flow diagram of patient selection and cohort formation.

A total of 160 patients were initially screened. Patients with driver mutations (EGFR, ALK, ROS1) and those with missing or unknown vaccination data were excluded. The final cohort consisted of 88 patients who received second-line nivolumab therapy and were categorized according to the number of mRNA COVID-19 vaccine doses (Figure 1).

### 2.2. Clinical and Pathological Variables

Baseline demographic and clinical characteristics were extracted from electronic medical records, including age at diagnosis, sex, ECOG performance status, histological subtype, smoking history, presence of comorbidities, baseline brain metastasis, metastatic sites, and PD-L1 expression status.

Progression-free survival following first-line therapy (PFS1) was defined as the time from initiation of first-line platinum-based chemotherapy to radiologically confirmed disease progression or death from any cause.

Tumor response and disease progression were assessed according to the Response Evaluation Criteria in Solid Tumors (RECIST) version 1.1 based on routine radiologic evaluations. Best overall response was classified according to RECIST v1.1 as complete response, partial response, stable disease, or progressive disease.

PD-L1 expression was assessed using tumor proportion score (TPS) and categorized as <1%, 1–49%, ≥50%, or unknown when unavailable.

Outcome Measures

Primary Endpoint

Progression-Free Survival 2 (PFS2)

Disease progression was assessed based on radiological imaging and clinical evaluation in routine clinical practice.

PFS2 was defined as the time from initiation of second-line ICI therapy (T0) to the occurrence of either second disease progression (PD2) during second-line systemic treatment or death from any cause, whichever occurred first.

Disease progression (PD2) was determined based on radiologic assessment according to RECIST v1.1 criteria documented in the medical records. Radiologic assessments were performed approximately every 8–12 weeks in routine clinical practice, or earlier if clinically indicated.

Patients without documented PD2 or death at the time of analysis were censored at the date of last follow-up.

PFS2 was selected as the primary endpoint because immune checkpoint inhibitors were not available as first-line treatment for advanced NSCLC in the study period in our country. Consequently, ICI therapy could only be initiated after documented progression on first-line chemotherapy. In this real-world setting, conventional PFS would not appropriately capture the treatment effect of ICI therapy. PFS2 therefore provides a more clinically meaningful measure of disease control following initiation of second-line immunotherapy.

### 2.3. Statistical Analysis

Continuous variables were expressed as median (interquartile range, IQR), and categorical variables as number and percentage. Because the primary exposure variable was defined as a binary grouping (0–1 vs. ≥2 mRNA doses), statistical comparisons were performed using two-group tests. Between-group comparisons were performed using the chi-square or Fisher’s exact test for categorical variables and the Mann–Whitney U test for continuous variables, as appropriate.

ECOG performance status was not included in multivariable models due to limited variability, as the vast majority of patients (97.7%) had ECOG 0–1.

Progression-free survival 2 (PFS2) was estimated using the Kaplan–Meier method and compared using the log-rank test. Median survival times with 95% confidence intervals (CIs) were reported. PFS2 was selected to reduce potential bias related to treatment sequencing and to better reflect real-world clinical practice in settings where immunotherapy is administered in later lines.

Univariable Cox regression analyses were initially performed to evaluate associations between individual covariates and PFS2. Multivariable Cox proportional hazards models were constructed, including covariates selected a priori based on established prognostic relevance in metastatic NSCLC (age, sex, smoking history, histology subtype, comorbidity status, baseline brain metastases, and PD-L1 tumor proportion score). Covariates included in the multivariable model were selected based on established prognostic relevance in metastatic NSCLC and availability within the retrospective dataset.

To minimize the risk of overfitting, the number of covariates was limited relative to the number of observed progression events. Specifically, 71 PFS2 events were observed, and the number of covariates included in the multivariable model was restricted to maintain an acceptable events-per-variable ratio. Multicollinearity was assessed using variance inflation factors (VIFs). Hazard ratios (HRs) with 95% confidence intervals were reported. The proportional hazards assumption was evaluated using graphical inspection and Schoenfeld residuals.

Potential effect modification between vaccination status and PD-L1 expression was explored through interaction testing. These interaction analyses were considered exploratory. Because vaccination practices evolved during the study period, potential calendar-time effects were considered during the interpretation of the results.

Among vaccinated patients, the interval (in days) between the last mRNA vaccine dose and nivolumab initiation (T0) was analyzed as a continuous variable in Cox regression to evaluate timing-related effects.

Sensitivity analyses excluding patients who received only one mRNA vaccine dose were performed to assess robustness.

No missing data were observed for the variables included in the analyses.

All analyses were conducted using IBM SPSS Statistics version 26.0 (IBM Corp., Armonk, NY, USA). A two-sided *p*-value < 0.05 was considered statistically significant. Median follow-up time was estimated using the reverse Kaplan–Meier method.

## 3. Results

Baseline demographic and clinical characteristics are summarized in Table 1. The median age at diagnosis was 66.0 years (IQR, 57.0–68.2), and the majority of patients were male (82.9%). Most patients had an ECOG performance status of 0–1 (97.7%). Adenocarcinoma was the most common histological subtype (44.3%), followed by squamous cell carcinoma (38.6%). Brain metastases at baseline were present in 27.3% of patients.

PD-L1 tumor proportion score (TPS) distribution was <1% in 32 patients (36.4%), 1–49% in 28 patients (31.8%), ≥50% in 8 patients (9.1%), and unknown in 20 patients (22.7%). All patients received nivolumab as second-line ICI therapy.

Baseline characteristics stratified by mRNA vaccination status are presented in Table 2. No statistically significant differences were observed between groups with respect to age, sex, histology, smoking status, comorbidity, or baseline brain metastasis (all *p* > 0.05).

**Figure 2:**
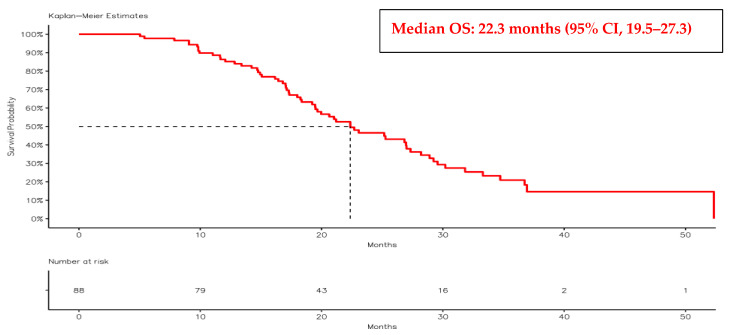
Kaplan–Meier curve illustrating overall survival (OS) in the study cohort.

**Figure 3:**
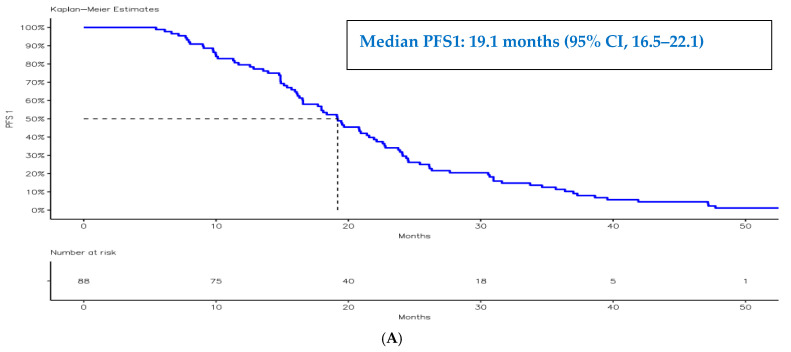

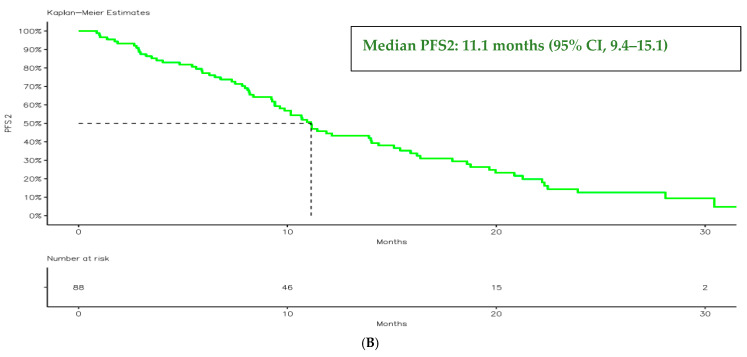
Kaplan–Meier curves illustrating progression-free survival following first-line therapy (PFS1) (**A**) and progression-free survival following second-line therapy (PFS2) (**B**).

Median follow-up time, estimated using the reverse Kaplan–Meier method, was **22.4 months** (calculated from nivolumab initiation to last clinical contact or death); the median OS for the entire cohort was 22.3 months (95% CI, 19.5–27.3). The median progression-free survival following first-line therapy (PFS1) was 19.1 months (95% CI, 16.5–22.1), while the median PFS2 was 11.1 months (95% CI, 9.4–15.1) (Figure 2 and Figure 3).

A total of 71 PFS2 events were observed in the study cohort.

**Figure 4:**
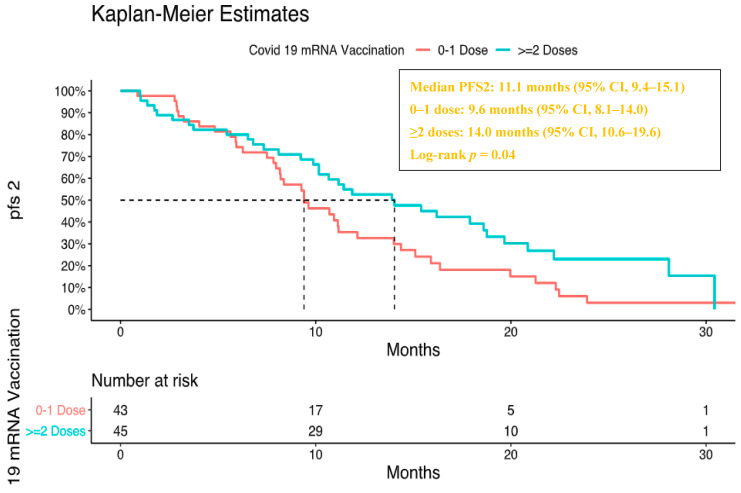
Kaplan–Meier curve showing progression-free survival 2 (PFS2) according to the number of COVID-19 mRNA vaccine doses.

A statistically significant difference in PFS2 was observed between patients who received 0–1 mRNA COVID-19 vaccine doses and those who received ≥2 doses (median PFS2: 9.6 vs. 14.0 months, log-rank *p* = 0.04) (Figure 4).

In univariable Cox proportional hazards analysis, receipt of ≥2 mRNA COVID-19 vaccine doses was associated with a significantly reduced risk of disease progression or death compared with 0–1 dose (HR, 0.60; 95% CI, 0.37–0.97; *p* = 0.03). This association remained significant after adjustment for age, sex, histology, smoking status, comorbidity, baseline brain metastasis, and PD-L1 TPS category (aHR, 0.52; 95% CI, 0.31–0.88; *p* = 0.01).

In multivariable analysis, non-adenocarcinoma histology (aHR, 1.91; 95% CI, 1.06–3.44; *p* = 0.02) and presence of brain metastasis at baseline (aHR, 1.97; 95% CI, 1.11–3.49; *p* = 0.02) were independently associated with shorter PFS2. PD-L1 TPS category demonstrated a significant overall association with PFS2 in multivariable analysis (global *p* = 0.03) (Table 3).

## 4. Discussion

In this single-center retrospective cohort of metastatic NSCLC patients treated with second-line nivolumab monotherapy, we observed that patients who had received ≥2 doses of mRNA COVID-19 vaccines within the 6 months prior to ICI initiation (T0) experienced significantly longer progression-free survival 2 (PFS2) compared with those with 0–1 doses. Multivariable Cox regression analysis demonstrated that this association remained statistically significant after adjusting for known prognostic factors, including age, sex, smoking history, histology subtype, comorbidity, baseline brain metastases, and PD-L1 tumor proportion score (TPS). Non-adenocarcinoma histology and presence of brain metastasis were independently associated with shorter PFS2, while PD-L1 TPS category demonstrated an overall association with PFS2 in the adjusted model.

The observed relationship between mRNA COVID-19 vaccination and improved second-line immunotherapy outcomes may be grounded in biologically plausible mechanisms. A recent high-impact study by Grippin and colleagues demonstrated that clinically available SARS-CoV-2 mRNA vaccines can sensitize tumours to immune checkpoint blockade (ICB) by inducing a robust type-I interferon (IFN-I) response, promoting activation of innate immune cells, enhancing CD8^+^ T-cell priming, and increasing tumoral PD-L1 expression in preclinical models and human cohorts [22]. The authors reported that receipt of SARS-CoV-2 mRNA vaccination within 100 days of initiating ICI was associated with improved overall survival in multiple retrospective cohorts, particularly in patients with immunologically “cold” tumours that typically respond poorly to checkpoint inhibition [22].

A growing body of commentary and follow-up literature has contextualized and extended these findings. Chi’s work in *Signal Transduction and Targeted Therapy* emphasizes that SARS-CoV-2 mRNA vaccines can transiently reset the tumour-immune interface, potentially converting immune-cold to more responsive environments favorable for PD-1/PD-L1 blockade [27]. Similarly, expert discussions have proposed that the potent and broad innate immune stimulation elicited by mRNA vaccines—beyond antigen specificity—may act as a systemic “primer” for adaptive antitumour responses when checkpoint inhibition is administered, aligning with the concept of immune sensitization in resistant tumour microenvironments [28]. Additional literature in the context of lung cancer vaccines discusses the potential for immune priming strategies to augment the efficacy of ICIs, supporting the broader concept that combining vaccination or immune stimulatory approaches with immunotherapy could overcome intrinsic resistance mechanisms [29].

These mechanistic insights are complemented by observational signals from other cohorts that examined the timing of mRNA vaccination relative to ICI initiation. However, a potential healthy-user bias should be acknowledged. Patients receiving multiple vaccine doses may represent a subgroup with better healthcare access, adherence, or overall baseline health status. Although major clinical variables were adjusted for, residual confounding cannot be completely excluded. Retrospective data suggest that receiving COVID-19 mRNA vaccines within a defined window before or around the start of immunotherapy is associated with improved survival outcomes across diverse patient populations, including NSCLC and melanoma [22,30]. Although these associations cannot establish causality, they provide further clinical signals consistent with the results of Grippin et al. and with our findings.

Residual confounding remains an inherent limitation of retrospective observational studies evaluating vaccination exposure. In addition to healthcare utilization patterns (healthy-user bias), unmeasured factors such as prior SARS-CoV-2 infection, baseline corticosteroid exposure, antibiotic use, and detailed disease-burden indicators may also influence immunotherapy outcomes. Although major clinical prognostic variables were included in the multivariable model, these factors could not be fully captured in this retrospective dataset.

The use of PFS2 as the primary endpoint in our study has clinical relevance in settings where first-line immunotherapy was not widely available during the study period. In such contexts, PFS1 may inadequately capture the clinical benefit contributed by second-line nivolumab, whereas PFS2 better reflects disease control attributable to later-line immunotherapy. Defining vaccination exposure as a fixed baseline variable using the 6-month window preceding ICI initiation was intended to capture a clinically relevant immune-priming interval and to avoid exposure misclassification after treatment start, thereby strengthening the inference regarding the temporal relationship between vaccination and immunotherapy outcomes [31]. Patients receiving only one mRNA vaccine dose were grouped with the unvaccinated group because the biologically relevant exposure of interest was the receipt of at least two recent mRNA doses, which may be required to induce a meaningful immune-priming effect.

Despite these strengths, several limitations warrant consideration. First, this study has a retrospective, single-center design, which may limit the generalizability of the findings and preclude definitive causal inference. Second, vaccination exposure was modeled as a fixed baseline variable defined prior to nivolumab initiation, and time-dependent exposure analyses were not performed. Although this approach reduces exposure misclassification after treatment initiation, immortal time bias related to post-treatment exposure classification was minimized. Third, residual confounding related to healthcare utilization patterns, access to care, or overall health behavior—including potential healthy-user bias among vaccinated individuals—cannot be completely excluded despite adjustment for major clinical covariates. Fourth, the PD-L1 tumor proportion score was unavailable for a subset of patients, which may have introduced additional heterogeneity in the multivariable analysis. Finally, radiological assessment intervals were determined according to routine clinical practice rather than a standardized protocol, which could potentially introduce variability in progression detection. These limitations highlight the need for prospective multicenter studies incorporating standardized imaging schedules, time-dependent exposure modeling, and integrated immune profiling to further clarify the relationship between mRNA vaccination and immunotherapy outcomes.

The study period (January 2023 to January 2026) coincided with evolving vaccination practices and changing epidemiological dynamics of SARS-CoV-2. Although vaccination status was defined relative to treatment initiation, potential residual confounding related to calendar time effects cannot be completely excluded.

Overall survival was analyzed descriptively. Because vaccination exposure was defined relative to the initiation of second-line nivolumab (T0), OS reflects cumulative variability arising from prior first-line treatment duration, response patterns, and underlying tumor biology. Consequently, OS may not isolate the immunotherapy-specific contribution of recent mRNA vaccination as precisely as PFS2 and should be interpreted within this context.

Prospective studies with standardized treatment and follow-up protocols, rigorous time-dependent exposure modeling, and robust immune profiling are necessary to validate the observed associations. From a clinical perspective, our findings align with the hypothesis that mRNA-mediated innate immune activation may enhance the therapeutic efficacy of PD-1 blockade in metastatic NSCLC, but they remain hypothesis-generating [22]. The integration of vaccination timing as a potential modulator of immunotherapy outcomes could inform future clinical trial design, including prospective evaluations of immune priming strategies, personalized sequencing of vaccines and ICIs, and investigations into biomarkers that predict benefit from such combinations [30].

## 5. Conclusions

In conclusion, our study demonstrates a significant association between prior mRNA COVID-19 vaccination and longer PFS2 in patients with metastatic NSCLC treated with second-line nivolumab. In light of mechanistic evidence from Grippin et al. and supportive signals from emerging follow-up literature, these observations contribute to a growing body of evidence suggesting that non-tumour-targeted mRNA vaccines may serve as systemic immune modulators capable of augmenting immune checkpoint blockade. Prospective, multicenter studies are required to determine whether mRNA vaccination can be intentionally leveraged to improve clinical outcomes in patients undergoing cancer immunotherapy.

## Figures and Tables

**Table 1:** Baseline Demographic and Clinical Characteristics.

**Age at diagnosis**, median (IQR)	66.0 (57.0–68.2)
Sex, n (%)	
Male	73 (82.9)
Female	15 (17.1)
ECOG performance status, n (%)	
0–1	86 (97.7)
≥2	2 (2.3)
Histology, n (%)	
Adenocarcinoma	39 (44.3)
Squamous	34 (38.6)
Other	15 (17.1)
Smoking status, n (%)	
Never	15 (17.1)
Former	44 (50.0)
Current	29 (32.9)
Comorbidity present, n (%)	47 (53.4)
Brain metastasis at baseline, n (%)	24 (27.3)
Metastatic sites, n (%) *	
Contralateral Lung	26 (29.5)
Adrenal Gland	15 (17.0)
Bone	30 (34.1)
Liver	8 (9.1)
Pleura	12 (13.6)
Mediastinal	35 (39.7)
PD-L1 tumor proportion score (TPS), n (%)	
<1%	32 (36.4)
1–49%	28 (31.8)
≥50%	8 (9.1)
Unknown	20 (22.7)
First-line chemotherapy regimen, n (%)	
Carboplatin + Paclitaxel	54 (61.4)
Gemcitabine + Cisplatin	17 (19.3)
Cisplatin + Pemetrexed	17 (19.3)
COVID-19 vaccination status, n (%)	
Unvaccinated/Inactivated vaccine only	18 (20.4)
mRNA vaccine only	14 (15.9)
Both inactivated + mRNA	56 (63.6)
Total number of COVID-19 mRNA vaccine doses, n (%)	
0–1 dose	43 (48.8)
≥2 doses	45 (51.2)

* Patients may have more than one metastatic site.

**Table 2:** Baseline Demographic and Clinical Characteristics According to mRNA Vaccination Status.

	0–1 Dose (n = 43)	≥2 Doses (n = 45)	*p*-Value
Age at diagnosis, median (IQR)	65.0 (56.5–68.5)	66.0 (58.0–68.0)	0.587
Sex, n (%)			0.923
Male	35 (81.4)	38 (84.4)	
Female	8 (18.6)	7 (15.6)	
Histology, n (%)			0.677
Adenocarcinoma	17 (39.5)	22 (48.9)	
Squamous	18 (41.9)	16 (35.6)	
Other	8 (18.6)	7 (15.6)	
Smoking status, n (%)			0.158
Never	9 (20.9)	6 (13.3)	
Former	17 (39.5)	27 (60.0)	
Current	17 (39.5)	12 (26.7)	
Comorbidity present, n (%)			0.131
No	16 (37.2)	25 (55.6)	
Yes	27 (62.8)	20 (44.4)	
Brain metastasis at baseline, n (%)			0.711
No	30 (69.8)	34 (75.6)	
Yes	13 (30.2)	11 (24.4)	

**Table 3:** Univariable and multivariable analyses of clinical and treatment-related factors associated with PFS2.

	Univariable		Multivariable	
Variable	HR (95% CI)	*p*-Value	aHR (95% CI)	*p*-Value
mRNA vaccine dose group (≥2 vs. 0–1)	0.60 (0.37–0.97)	**0.03**	0.52 (0.31–0.88)	**0.01**
Age (per year increase)	1.01 (0.98–1.04)	0.30	1.03 (0.99–1.06)	0.09
Sex (Male vs. Female)	0.61 (0.33–1.12)	0.11	0.45 (0.14–1.42)	0.14
Histology (Non-adenocarcinoma vs. Adenocarcinoma)	1.15 (0.71–1.86)	0.55	1.91 (1.06–3.44)	**0.02**
Smoking status (Ever vs. Never)	0.59 (0.31–1.11)	0.10	1.03 (0.32–3.29)	0.95
Comorbidity present (Yes vs. No)	1.24 (0.37–2.00)	0.37	0.87 (0.51–1.49)	0.62
Brain metastasis at baseline (Yes vs. No)	1.59 (0.94–2.68)	0.08	1.97 (1.11–3.49)	**0.02**
PD-L1 TPS category (overall)		0.65		**0.03**

Abbreviations: HR, hazard ratio; aHR, adjusted hazard ratio CI, confidence interval;TPS, tumor proportion score; PFS2, progression-free survival 2. PD-L1 TPS was categorized as <1%, 1–49%, ≥50%, or unknown, with <1% as the reference category.

## Data Availability

The datasets generated and analyzed during the current study are available from the corresponding author upon reasonable request.

## Abbreviations

The following abbreviations are used in this manuscript:

aHR	adjusted hazard ratio
CI	confidence interval
ECOG	Eastern Cooperative Oncology Group
HR	hazard ratio
ICB	immune checkpoint blockade
ICI	immune checkpoint inhibitor
IFN-I	type I interferon
IFN-γ	interferon gamma
IL-12	interleukin 12
IQR	interquartile range
mRNA	messenger RNA
NSCLC	non-small cell lung cancer
OS	overall survival
PD-1	programmed cell death protein 1
PD2	second disease progression
PD-L1	programmed death-ligand 1
PFS1	progression-free survival following first-line therapy
PFS2	progression-free survival following second-line therapy
T0	initiation date of second-line immune checkpoint inhibitor therapy
TPS	tumor proportion score
VIF	variance inflation factor
